# Larval time-to-hatch and insecticide resistance in the major malaria vector *Anopheles gambiae* from Ghana

**DOI:** 10.1186/1475-2875-9-S2-O5

**Published:** 2010-10-20

**Authors:** Basil D Brooke, Maria L Kaiser, Lizette L Koekemoer, Maureen Coetzee, Richard H Hunt

**Affiliations:** 1 Vector Control Reference Unit, National Institute for Communicable Diseases, NHLS, Private bag X4, Sandringham, 2131, South Africa; 2 Malaria Entomology Research Unit, School of Pathology of the University of the Witwatersrand and the National Health Laboratory Service, Johannesburg, South Africa; 3 School of Animal, Plant and Environmental Sciences, University of the Witwatersrand, Private Bag X3, University of the Witwatersrand, Johannesburg, South Africa

## Background

Malaria is holoendemic in Ghana. The effectiveness of insecticide based vector control methods is hampered there by the development of insecticide resistance. Resistance to multiple classes of insecticide has previously been detected in a population of the major malaria vector *Anopheles gambiae* in the Obuasi region of Ghana. The establishment of a laboratory colony (GAH) using wild *An. gambiae* S form material from Obuasi has enabled characterization of multiple insecticide resistance in the GAH colony as well as an appraisal of the effect of staggered larval time-to-hatch on the assortment of resistance phenotypes.

## Materials and methods

Larval-time-to-hatch, characterized as either early (within four days of oviposition) or late hatch (four days or more post oviposition), was assessed in the GAH colony by selecting time-to-hatch sub-colonies. Crosses between the early and late hatch sub-colonies as well as intercrosses and subsequent back-crosses to the parental sub-colonies were conducted in an effort to establish the genetic basis for larval time-to-hatch. Insecticide resistance in the baseline colony as well as in the selected sub-colonies was characterized using standard WHO bioassays, synergist exposure assays and allele specific PCR assays designed to genotype loci carrying known insecticide resistance associated mutations.

## Result

The GAH colony shows resistance to all classes of insecticide currently available for use in public health. Mechanisms of resistance to pyrethroids in GAH include monooxygenase and esterase mediated detoxification coupled with the L1014F *kdr* mutation. Resistance to DDT associated particularly closely with the assortment of L1014F *kdr.* Resistance to dieldrin associated closely with the *rdl* mutation while resistance to the carbamate bendiocarb associated closely with the *ace-1* mutation. Cross-mating between the time-to-hatch phenotypes, supported by hybrid intercrosses and back-crossing of hybrids to the parental strains, gave ambiguous results suggesting that this genetic component may be multi-factorial. There was significant variation in the expression of insecticide resistance between the time-to-hatch phenotypes, suggesting that selection for time-to-hatch inadvertently affected the frequencies of those factors controlling insecticide resistance.

## Conclusion

Variation in the expression of insecticide resistance in association with selection for larval time-to-hatch may induce enhanced adaptive plasticity as a consequence of pleiotropy. In this scheme cohorts of mosquitoes are able to complete their aquatic life stages in a variable breeding environment using staggered larval time-to-hatch, giving rise to an adult population with enhanced variation in the expression of insecticide resistance. Such enhanced variation offers a wider phenotypic platform for the selection of resistance to insecticides.

